# Foundational biodiversity effects propagate through coastal food webs via multiple pathways

**DOI:** 10.1002/ecy.3796

**Published:** 2022-07-27

**Authors:** Aaron P. Ramus, Jonathan S. Lefcheck, Zachary T. Long

**Affiliations:** ^1^ Department of Biology and Marine Biology University of North Carolina Wilmington Wilmington North Carolina USA; ^2^ Tennenbaum Marine Observatories Network MarineGEO, Smithsonian Environmental Research Center Edgewater Maryland USA

**Keywords:** biodiversity–ecosystem functioning, coastal marine ecosystems, epifaunal invertebrates, foundation species, niche complementarity, nonnative species, plant diversity effects, structural equation modeling

## Abstract

Relatively few studies have attempted to resolve the pathways through which the effects of biodiversity on ecosystem functioning cascade from one trophic level to another. Here, we manipulated the richness of habitat‐forming seaweeds in a western Atlantic estuary to explore how changes in foundation species diversity affect the structure and functioning of the benthic consumer communities that they support. Structural equation modeling revealed that macroalgal richness enhanced invertebrate abundance, biomass, and diversity, both directly by changing the quality and palatability of the foundational substrate and indirectly by increasing the total biomass of available habitat. Consumer responses were largely driven by a single foundational seaweed, although stronger complementarity among macroalgae was observed for invertebrate richness. These findings with diverse foundational phyla extend earlier inferences from terrestrial grasslands by showing that biodiversity effects can simultaneously propagate through multiple independent pathways to maintain animal foodwebs. Our work also highlights the potential ramifications of human‐induced changes in marine ecosystems.

## INTRODUCTION

Current understanding of biodiversity‐ecosystem functioning (BEF) relationships has largely been advanced by studies focused within a single trophic level, typically involving primary producers and, more specifically, terrestrial grasses and forbs (Cardinale et al., 2011). Though these manipulations have brought us significant insight into the general relationship between the number of species, their taxonomic, functional, and phylogenetic identities, and ecosystem processes such as primary production, nutrient cycling, and decomposition (Cardinale et al., 2012), comparatively fewer studies have examined how biodiversity effects within a given trophic level propagate throughout multitrophic food webs (i.e., “vertical” diversity sensu Duffy, 2002). Those that do can be broken down into two categories, studies that manipulate either (i) consumer diversity (e.g., herbivores and predators) and measure consumption and loss of plant biomass (Bruno & O'Connor, 2005; Douglass et al., 2008; O'Connor & Bruno, 2007) or (ii) resource diversity (e.g., nutrients or detritus, aka “brown food webs”) and quantify effects on consumer standing stock and community structure (Srivastava et al., 2009).

Since the earliest work on niche theory by Grinnell (1917), habitat has also been recognized as a key limiting resource in natural communities, and ecologists have increasingly begun to explore how the presence and diversity of habitat‐forming foundation species can influence the structure and function of adjacent trophic levels from the “bottom up” (i.e., a resource‐based view; Borst et al., 2018, Thomsen et al., 2010, van der Zee et al., 2016). For example, previous studies in grasslands showed that plant diversity could enhance the biomass and diversity of terrestrial arthropod communities through both direct (Ebeling et al., 2018; Scherber et al., 2010) and indirect pathways, generally by enhancing plant biomass (Borer et al., 2012; Hertzog et al., 2016). More recent experiments demonstrate that this direct link between the species richness of grasses and trees and herbivore abundances is mediated by the functional, morphological, and chemical attributes of plants (Schuldt et al., 2019). Thus, there exist several potential mechanisms for the propagation of positive diversity effects from foundation species to adjacent trophic levels (Long et al., 2007). These include changing either the quality (Schuldt et al., 2019) or the quantity of habitat (i.e., standing biomass), or a combination of the two (Borer et al., 2012; Hertzog et al., 2016). There are two corollaries to these options: first, when habitat formers also serve as a food resource, palatability becomes a consideration (Schuldt et al., 2019); second, when foundation species also provide habitat for higher trophic levels, predators can suppress herbivores through top‐down forces (Haddad et al., 2009).

Disentangling these (potentially nonexclusive) pathways by which foundational biodiversity effects propagate through food webs is a critical challenge because ecosystems worldwide are becoming increasingly threatened by anthropogenic activities that are resulting in local extinctions and introductions of foundation species (Barnes et al., 2014; Byrnes et al., 2007; Wardle et al., 2011). Coastal ecosystems in particular provide an exemplary testbed for unraveling how losses and gains of key habitat‐forming species, such as cordgrasses (Gedan et al., 2009; Silliman & Bertness, 2004), kelps (Krumhansl et al., 2016; South et al., 2017), mangroves (Fourqurean et al., 2010; Smith et al., 2018), seagrasses (Posey, 1988; Waycott et al., 2009), and seaweeds (Thomsen et al., 2016; Walker & Kendrick, 1998), alter the diversity and structure of complex natural food webs they support. Yet much of our understanding of vertical BEF relationships in the ocean to date has been derived from top‐down manipulations of invertebrate assemblages in seagrass ecosystems (Duffy, 2006; Duffy et al., 2007). Though researchers have recently begun to embrace the idea of foundation species diversity and its effects on habitat provisioning in marine ecosystems (e.g., Alsterberg et al., 2017; Hughes et al., 2010; Hughes & Stachowicz, 2011; Jochum et al., 2012), including intraspecific diversity (Reynolds et al., 2016), this topic remains an open area of research that constitutes a significant gap in our understanding, particularly in comparison to terrestrial ecosystems (Gamfeldt et al., 2015; Stachowicz et al., 2007).

Early ecology was spurred by numerous studies on macroalgae occurring in rocky inter‐ and subtidal communities (Connell, 1972; Lubchenco & Menge, 1978; Paine, 1974). Despite traditionally being considered less “desirable” when replacing other foundation species, like corals and seagrasses (e.g., Hughes, 1994), these macroalgae represent a diverse consortium of species whose habitat value is now becoming increasingly recognized (Fulton et al., 2020; Metzger et al., 2019; Olafsson, 2017; Rasher et al., 2020). Moreover, whereas the majority of biodiversity research has been conducted in terrestrial grassland using replacements of species representing a few closely related families (e.g., grasses and forbs), macroalgae are phylogenetically more diverse—the Rhodophyta (reds), Phaeophyta (browns), and Chlorophyta (greens) represent distinct, divergent evolutionary lineages, exhibit characteristic functional traits that reflect their underlying structural variation (Cappelatti et al., 2020; Mauffrey et al., 2020; Steneck & Dethier, 1994), and often make up a large part of biomass in coastal systems (Duarte et al., 2022; Olafsson, 2017). Macroalgae are also easily transported, and macroalgal invasions are becoming increasingly prevalent worldwide (Schaffelke et al., 2006), with unknown implications for diversity‐functioning relationships. Macroalgae therefore provide an ideal model system that is readily accessible, is easily manipulated, and exhibits high compositional turnover rates, allowing researchers to conveniently measure the response of consumers under realistic conditions, including the presence of invaders, a topic that has received comparatively less research attention in the BEF literature.

In this study, we manipulated algal species richness over several months and measured the consequences for the structure and functioning of invertebrate consumer communities that rely on foundational macroalgae for both shelter and food (Duffy & Hay, 1991). These consumers comprise naturally abundant herbivorous grazers, such as amphipods and gastropods, and their predators, including many juvenile decapod crustaceans of ecological and economic importance. We first applied structural equation modeling to investigate the flow of direct effects from macroalgal richness to invertebrate richness and biomass, and whether this relationship was indirectly mediated by macroalgal wet mass. We next searched for evidence of increased performance in species mixtures relative to monoculture (i.e., overyielding) and whether mixtures outperformed the “best” monoculture (i.e., transgressive overyielding), as well as how macroalgal species identity and richness influenced invertebrate community composition. We then used traditional partitioning of the net effect of biodiversity into its component complementarity and selection effects following Loreau and Hector (2001) in order to understand the mechanisms mediating secondary production of consumers. Here, the selection effect quantifies the degree to which species with particular traits dominate in mixtures, whereas the complementarity effect quantifies the degree to which niche partitioning or positive interactions allow species to more efficiently utilize resources in mixtures (Long et al., 2007). Finally, we addressed the question of what role a nonnative species might play in driving the observed relationships.

We expected that (i) macroalgal species identity would influence secondary production metrics more strongly than species richness (Bates & DeWreede, 2007; Best et al., 2014; Gustafsson & Boström, 2009; Parker et al., 2001); (ii) complementarity effects would be positive and selection effects would be slightly negative, resulting in an overall positive net biodiversity effect on secondary production (following Boyer et al., 2009; Bruno et al., 2005, 2006; Gustafsson & Boström, 2011; Long et al., 2007); and (iii) shared evolutionary history among congenerics would result in few differences among treatments containing the native and nonnative *Gracilaria* species (Cardinale et al., 2011; Gan et al., 2019; Thomsen et al., 2014).

## METHODS

### Field experiment

This study builds off the design of our previous work in this system to analyze novel data, and we briefly describe the methodologies used in what follows (see Ramus & Long, 2016 for full details). In 2012, we used four species of macroalgae—*Codium fragile*, *Gymnogongrus griffithsiae*, *Gracilaria tikvahiae*, and the nonnative *Gracilaria vermiculophylla*—that occur in abundance on the rock revetment located within Zeke's Island National Estuarine Research Reserve (NOAA/NERRS; 33.9544, −77.9488, North Carolina). From these four species we generated seven treatments that represent three levels of richness, including four monocultures, two distinct three‐species mixtures that differed only by an inclusion of either the native or nonnative *Gracilaria* (in combination with *Codium* and *Gymnogongrus*), and a polyculture of all four species.

Beginning in June 2012, we collected experimental macroalgae from the rock revetment, submerged in freshwater, and manually abraded to remove epifauna and other associated organisms. Individual thalli were centrifuged to remove excess water and weighed to within ±1 g of predetermined values: 15 g for monocultures, 5 g for three‐species mixtures, and 4 g for the complete mixture, to ensure initial wet biomass was held constant across treatments (i.e., a substitutive design). We constructed replicated (*n* = 5) experimental fleshy macroalgae communities in the treatment assigned to each block by attaching thalli to 30 × 50‐cm black plastic screens (mesh size = 1 cm) using cable ties. To account for destructive sampling and replacement of developing communities each week (as described in the next paragraph), we replicated the assigned treatment in each quadrant of the screen (the position of individual species within multispecies treatments was determined haphazardly within each quadrant). Each mesh screen was anchored to the top surface of a 10 × 20 × 40‐cm concrete cinderblock that had been selected as an experimental substrate to mimic the rock revetment. We deployed the 35 concrete blocks subtidally on the bottom (i.e., below the mean low water line) spaced at 1‐m intervals in a randomized order parallel to, and 2 m distant from, the rock revetment. A schematic of our experimental design is given in Appendix S1: Figure S1.

After allowing the experimental macroalgal communities developed in situ for 8 weeks (and thus become more resolved), we destructively sampled and replaced one quadrant (i.e., one‐quarter) of the community developing on each screen by collecting thalli of each species individually and placing them along with their associated organisms into separate zip‐top bags. Quadrants within each screen were sampled sequentially such that the invertebrate community in each was allowed to develop in situ for at least 4 weeks. We then replaced the treatment in the sampled quadrant with appropriate defaunated thalli and returned the concrete block to its location on the bottom. Although the blocks were lifted from the water while replacing the sampled quadrant, this procedure was consistently applied across all replicates and to communities that are routinely subject to wave action and occasional exposure to air due to tides. All samples were collected (and treatments replaced) within a 24‐h period each week and stored on ice for transport. In the laboratory, macroalgae were rinsed in freshwater and shaken for ∼1 min to remove invertebrates, which were captured in a 500‐μm sieve and preserved in 75% ethanol. The final wet biomass of recovered macroalgae was determined after centrifugation to remove excess water. Invertebrates were identified to broad taxonomic groupings (typically family level) and enumerated under a stereomicroscope (∼18×) (Nikon SMZ800). The total biomass of invertebrates in each sample was determined with a precision balance after drying at 60°C for ≥72 h. We excluded eight replicates where the macroalgae were lost between deployment and retrieval, for a total of *n* = 132 replicates (5 replicates × 7 treatments × 4 weeks) for our final analysis.

### Statistical analysis

To link the responses between primary producers and heterotrophic invertebrates, we employed structural equation modeling (SEM), a technique that allows the investigator to assess hypothesized causal relationships across a network of potentially interconnected variables. We followed the example of previous studies (Borer et al., 2012; Ebeling et al., 2014; Joern & Laws, 2012; Scherber et al., 2010) when devising our initial model (Appendix S1: Figure S2), which we describe briefly. First, we expected that macroalgal richness would enhance macroalgal wet mass, as demonstrated in previous work (Boyer et al., 2009; Bruno et al., 2005, 2006). Second, we expected macroalgal richness to directly influence invertebrate community properties (i.e., abundance, biomass, and richness), reflecting changes in habitat quality that arise from the differing morphologies of the seaweed species. Third, we expected that macroalgal wet mass would enhance invertebrate abundance, biomass, and richness by providing more habitat or food resources. Finally, we expected that invertebrate richness would enhance invertebrate biomass. Because both richness and biomass are known to correlate strongly with abundance, we included abundance as a covariate in our model as a form of statistical control.

**FIGURE 1:**
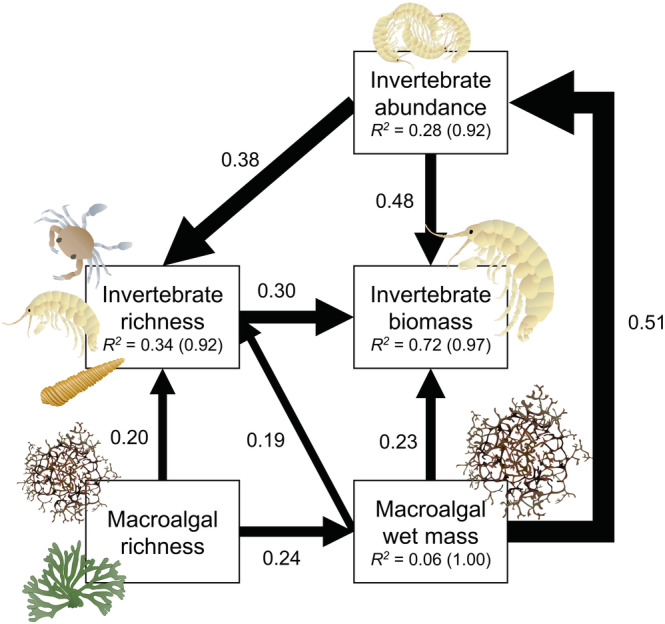
Final observed structural equation model relating experimentally manipulated macroalgal richness to properties of both macroalgae and associated invertebrate consumer communities (see Appendix S1: Figure S2 for the null‐hypothesized model). Arrows represent directed effects (i.e., flow of causality from one variable to another). Standardized regression coefficients are shown next to the arrows in units of SD of mean, such that they can be compared fairly across response variables of differing units. Arrow widths are scaled by the standardized coefficients (Appendix S1: Table S1). Marginal (fixed effects only) and conditional (fixed + random effects) *R* ^2^ values are also reported for each response variable.

Because our replicates are spatially and temporally autocorrelated, we employed linear mixed‐effects models (Pinheiro et al., 2021) to implement a nested varying‐intercept, fixed‐slope structure of an experimental block within a week. To satisfy the model assumptions of normality of errors, we visually assessed response variables and log_10_‐transformed macroalgal wet mass, invertebrate dry mass, and invertebrate richness. The individual linear mixed‐effects models were summarized in a single structural equation model using the piecewiseSEM package (Lefcheck, 2016). Because our model was saturated (i.e., there are no missing linkages between variables), we were unable to obtain a traditional goodness‐of‐fit statistic. Instead, we calculated marginal (fixed effects only) and conditional (fixed + random effects) *R* ^2^ values to assess the validity of the model (Nakagawa et al., 2017). We also used the sum of the individual likelihoods of the component models to be able to conduct model comparisons using AIC (Shipley & Douma, 2020). A useful property of the standardized regression coefficients (reported in Figure 1 and Appendix S1: Table S1) is that they can be multiplied to obtain the strength of the indirect effect. Finally, because pooled data yielded the same results qualitatively (Ramus et al., 2022, code 1), we present here our original analysis on unaggregated data.

To visualize the differences in invertebrate community composition among treatments, we used nonmetric multidimensional scaling (NMDS) plots that reduced the multivariate community matrix into a reduced set of dimensions for easier visualization. NMDS plots were based on the unweighted species abundances in each treatment over the final 4 weeks. Differences in invertebrate community composition among macroalgal treatments were assessed using a permutational multivariate analysis of variance, or PERMANOVA (Oksanen et al., 2020), with 999 permutations. We conducted pairwise planned contrasts to determine which macroalgal treatments differed in invertebrate community composition.

We partitioned the net effect of macroalgal species identity and richness into its component complementarity and selection effects and all values were square‐root‐transformed with the original sign preserved following Loreau and Hector (2001). We used *t*‐tests to determine whether net biodiversity, complementarity, and selection effects differed from zero. Separate analyses were conducted for each abundance, biomass, and richness and the different mixture types (three spp. native, nonnative, and four species). All analyses were conducted in R version 4.1.0 (R Core Team, 2021).

## RESULTS

Our structural equation model revealed significant bottom‐up pathways from the experimental macroalgal assemblages to the naturally recruiting consumer community (Figure 1 and Appendix S1: Table S1). First, as expected from previous experiments, macroalgal richness significantly enhanced standing wet mass (*p* = 0.005). In turn, both macroalgal richness and wet mass directly increased invertebrate richness (*p* = 0.007 and *p* = 0.031, respectively). Additionally, macroalgal richness indirectly enhanced invertebrate richness by increasing macroalgal wet mass. In the case of macroalgal effects on invertebrate richness, the directed path between macroalgal richness (β_std_ = 0.20) was approximately four times stronger than the indirect effect through wet mass (β_std_ = 0.24 × 0.19 = 0.05), suggesting that the animals are responding primarily to the experimental manipulation of macroalgal richness rather than the consequences of this manipulation for algal productivity.

Invertebrate biomass did not respond directly to the experimental manipulation of macroalgal richness (*p* = 0.538), but rather indirectly through the effects of increased macroalgal wet mass. Together, macroalgal wet mass increased invertebrate biomass directly (*p* < 0.001) and indirectly by increasing the richness of invertebrate taxa (*p* < 0.001). As previously, the direct effect (β_std_ = 0.23) was stronger than the indirect one (β_std_ = 0.19 × 0.30 = 0.06). An equally valid alternative configuration of our model is that invertebrate richness is increased by increasing invertebrate biomass. However, comparison of these two configurations revealed that the path from invertebrate richness to biomass was more likely than the reverse (AIC = 1344.5 vs. 1350.4). Finally, as expected, invertebrate abundance was strongly associated with both invertebrate biomass (*p* < 0.001) and richness (*p* < 0.001), as well as macroalgal wet mass (*p* < 0.001). The latter constituted the strongest linkage recovered in our analysis (Appendix S1: Table S1), reflecting the reliance of these small organisms on the total availability of habitat.

**FIGURE 2:**
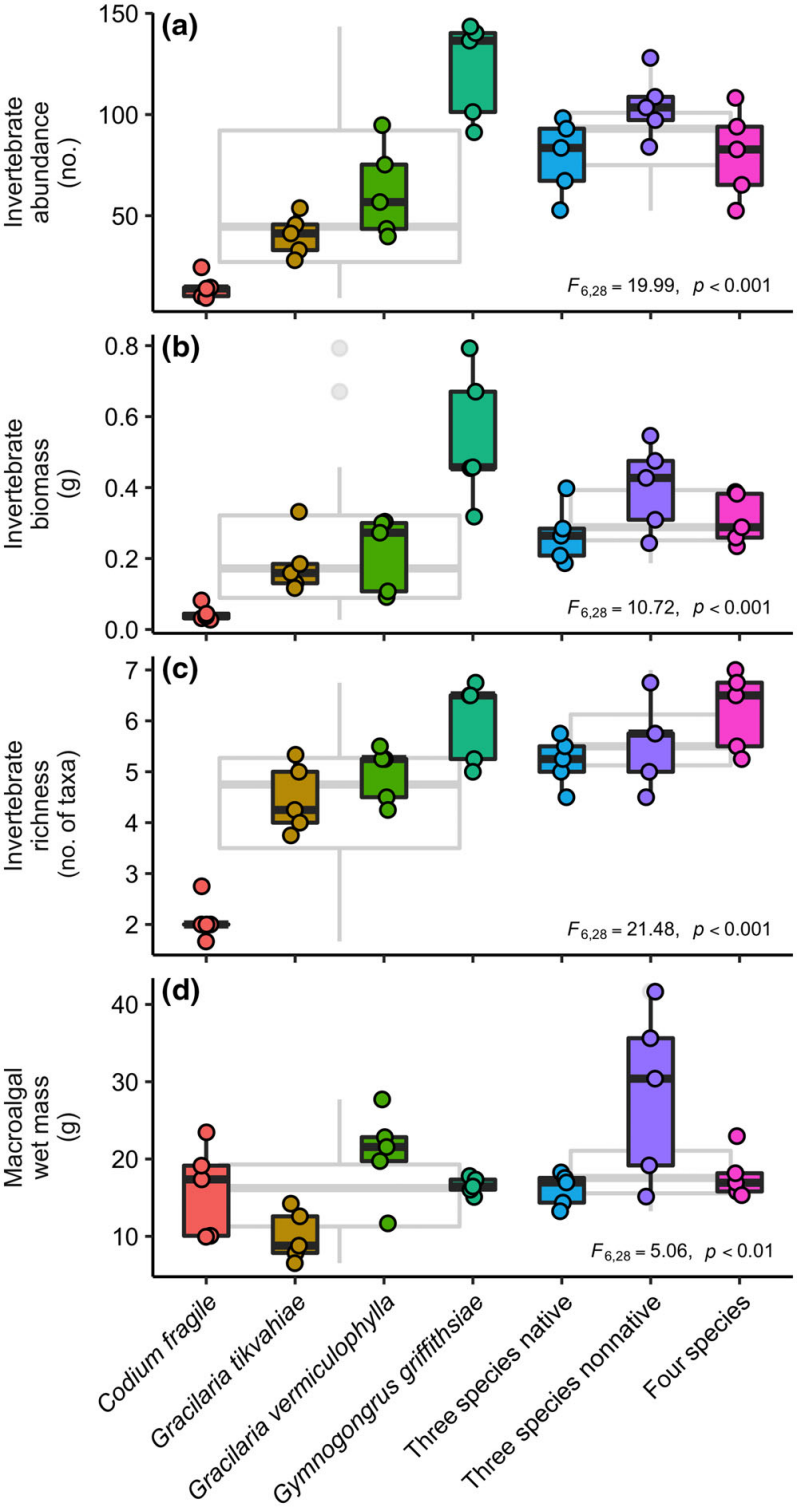
Effects of macroalgal species identity and richness on metrics of primary and secondary production. Invertebrate (a) abundance, (b) dry biomass, and (c) taxonomic richness, and (d) macroalgal wet mass. Points are the time‐averaged response of each replicate over the final 4 weeks (*n* = 5 for all treatments). Colors correspond to the seven experimental macroalgal treatments (see Tukey's honestly significant difference test for the results of post hoc analysis). Gray underlying boxplots represent pooled response of all monocultures and polycultures, respectively (see *t*‐tests for comparisons of means). The results of one‐way analyses of variance (ANOVAs) are shown near the margin of each panel.

Overyielding was consistently observed—the average mixture performed better than the average monoculture (Figure 2, gray boxes, *p* < 0.05 in all cases except invertebrate biomass, *t*‐tests)—but it was not considered transgressive because the average mixture did not perform better than the single‐species treatment for *Gymnogongrus* in any circumstance. Among the three mixtures, there were no differences in invertebrate abundance, biomass, and richness (Figure 2, *p* ≥ 0.251, Tukey's honestly significant difference [HSD] test), and macroalgal wet mass differed in only one case between the native and nonnative *Gracilaria* (*p* = 0.024, Tukey's HSD).

**FIGURE 3:**
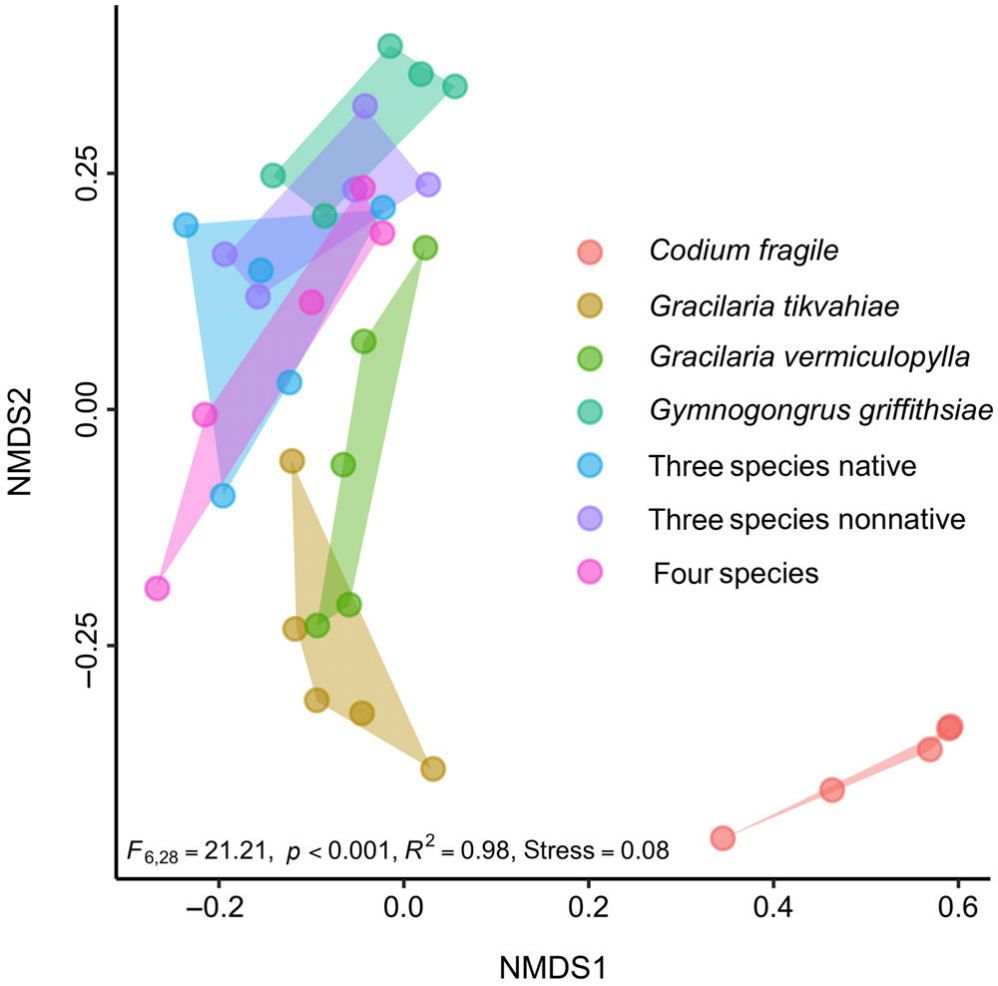
Nonmetric multidimensional scaling (NMDS) plot showing composition of invertebrate consumer community that colonized each experimental macroalgal treatment. Colors correspond to the seven experimental macroalgal treatments. Points represent the *n*‐dimensional response of each replicate and were calculated from the mean consumer abundance after time averaging over the final 4 weeks. Shaded hulls indicate the multidimensional space occupied by the invertebrate community in each treatment (see Appendix S1: Table S2 for results of pairwise planned contrasts). The results of permutational multivariate analysis of variance (PERMANOVA) based on the unweighted species abundances in each treatment are shown near the lower left margin (*n* = 5 for all treatments).

Similarly, our analysis of community composition revealed differences among the four habitat‐forming seaweeds in terms of the invertebrate assemblages associated with each (Figure 3). This may have occurred because non‐xanthid crabs were, on average, most prevalent on *Codium* and *Gracilaria tikvahiae*, and isopods and shrimps were more than twice as common on *Gracilaria vermiculophylla* than on *Gymnogongrus*. While the composition of recruiting consumers in *Gymnogongrus* did not differ from any of the mixtures (*p* ≥ 0.36, all cases; Appendix S1: Table S2), it did differ from *Gracilaria tikvahiae* (*F*_1,33_ = 6.49, *p* = 0.02), *Gracilaria vermiculophylla* (*F*_1,33_ = 3.23, *p* = 0.05), and *Codium* individually (*F*_1,33_ = 26.05, *p* = 0.01, Figure 3).

**FIGURE 4:**
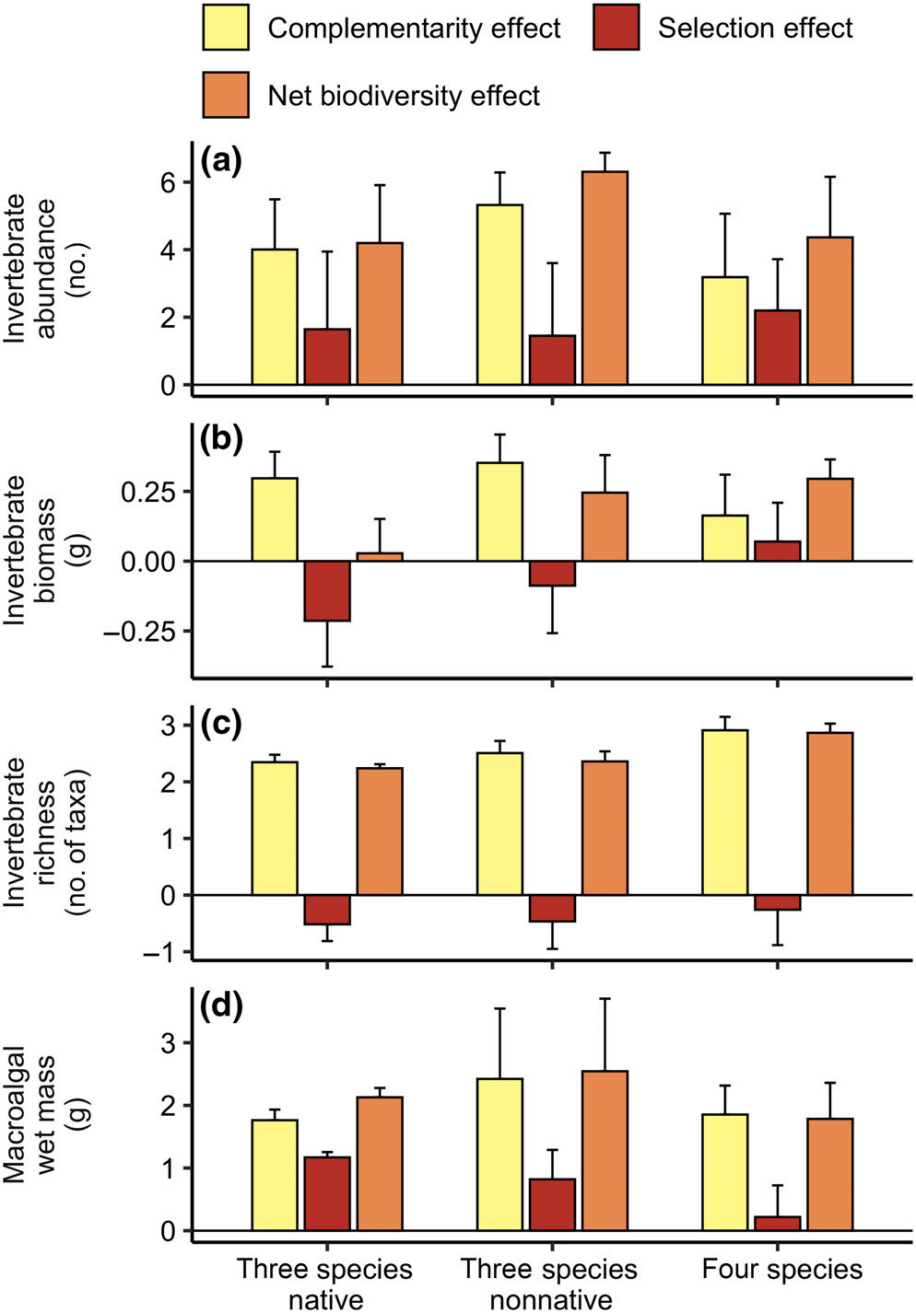
Net biodiversity effect partitioned into its component complementarity and selection effects for each response variable (rows) in multispecies treatments. (a) Invertebrate abundance, (b) biomass, (c) taxonomic richness and (d) macroalgal wet mass. Colors correspond to the three biodiversity effect components as indicated in the legend. Points were calculated from time‐averaged responses of each replicate over the final 4 weeks (*n* = 5 for all treatments), and all values were square‐root‐transformed with the original sign preserved (Loreau & Hector, 2001). Axis scales for biodiversity effect components are equivalent for each response variable (within rows), but not across response variables (among rows). Responses above the black line at 0 are positive, whereas those below are negative (see Appendix S1: Table S3 for results of *t*‐tests).

Indeed, partitioning of the net biodiversity effect revealed generally positive complementarity that was equal to the selection (identity) effect for macroalgal wet mass, invertebrate abundance, and invertebrate biomass in most multispecies treatments (Figure 4a,b,d), but for invertebrate richness, complementarity was the sole driver of the positive biodiversity effect (Figure 4c; Appendix S1: Table S3).

## DISCUSSION

In this in situ experimental manipulation of a nearshore benthic ecosystem, we showed that macroalgal richness can simultaneously influence the invertebrate community recruiting to this habitat through multiple pathways, including indirectly through enhanced macroalgal wet mass. These results corroborated earlier findings in terrestrial grasslands that demonstrate the capacity of direct and indirect pathways from species‐rich foundational communities to percolate through the developing food web (Borer et al., 2012; Scherber et al., 2010), although these have rarely been demonstrated in tandem (but see Hertzog et al., 2016). Nevertheless, this is the first example of this phenomenon in a relatively simple system of marine macroalgae occupying different phyla, which utilized four common species (including the nonnative *Gracilaria vermiculophylla*), compared to 16–60 species of grassland plants in previous studies. Moreover, we showed that the diversity effects recovered in our overarching analysis were largely driven by a single species, *Gymnogongrus griffithsiae*, although in the case of consumer richness, the other four species were generally complementary in promoting the recruitment of different consumers.

Our findings likely reflect perceived differences in the value of macroalgal species as shelter and food by most consumer species, arising from a complex set of trade‐offs involving morphological (structural) complexity, nutritional quality, and plant chemical defenses (Best et al., 2014; Erickson et al., 2006; Gan et al., 2019; Machado, Ferreira, & Leite, 2019; Parker et al., 2001; Wernberg et al., 2013). For example, *Gymnogongrus* is morphologically more complex than either *Gracilaria* species, which are nearly equivalent in complexity; all three species are, however, substantially more complex than *Codium*, which is structurally rather simple in form (Gan et al., 2019; Ramus & Long, 2016; Steneck & Dethier, 1994). Moreover, the nutritional content of *Codium* and *Gracilaria* species is similar to and likely to be greater than *Gymnogongrus* (Berke et al., 2020; Duffy & Hay, 1994; Hay et al., 1987). Finally, *Codium* and *Gracilaria* species are chemically defended by antinutritional compounds (Duffy & Hay, 1994; Hay et al., 1987), whereas *Gymnogongrus* is suspected to be mechanically defended by high silican or calcium content. Taken together, the natural history of these seaweeds suggests that *Gymnogongrus* most likely provides a superior habitat, but not a food resource, for associated organisms, an inference supported by the strong linkages between macroalgal wet mass and invertebrate richness, biomass, and particularly abundance in our structural equation model (Figure 1).

The dominance of *Gymnogongrus* for many community properties speaks to the major role of plant identity effects in structuring consumer communities and the general lack of evidence for strong complementarity in marine systems (Gamfeldt et al., 2015; Stachowicz et al., 2007). Our results are consistent with past studies showing that plant species and functional group richness effects are generally weak in comparison to species identity and composition, which have ecologically significant effects on primary and secondary production in epifaunal consumer communities (Bates & DeWreede, 2007; Best et al., 2014; Gustafsson & Boström, 2009; Parker et al., 2001) and terrestrial grasslands (Borer et al., 2012; Haddad et al., 2009; Siemann et al., 1998). One explanation is that larger species pools, like those found in terrestrial studies, provide more opportunities for true complementarity to arise (Gamfeldt et al., 2015). Nevertheless, most foundational marine habitats are monocultures (e.g., temperate seagrasses, oysters, and some salt marshes) dominated by one or few species, making our manipulation realistic in comparison to natural systems.

The role of high‐performing species does not necessarily preclude the potential for biodiversity effects (Lefcheck et al., 2021). Indeed, partitioning of the net biodiversity effect revealed that, despite overall strong identity effects, complementarity was strongest for consumer richness (Figure 4). Thus, although *Gymnogongrus* appears best suited to produce lots of (complex) habitat (Figure 2a), the other macroalgal species could and do attract representatives of species less commonly associated with the dominant macroalga, despite their potentially low contributions to abundance and biomass. This result could be due to the aforementioned differences in palatability, although the lack of transgressive overyielding would suggest that there is no evidence for spillover effects, for example, if consumers were to inhabit *Gymnogongrus* and forage on the other seaweeds in mixture. Other plausible drivers could be differences in the quantity or quality of macroalgal‐associated periphyton, which constitute the primary food sources for these invertebrate consumers (Duffy & Hay, 1991), or even within‐habitat trophic interactions, since the crabs and shrimps associated with seaweeds other than *Gymnogongrus* tend to be larger and more mobile, on average. Therefore, they might be attracted to and consequently deplete other smaller crustaceans that they are known to consume (Douglass et al., 2011), as shown previously in grasslands where terrestrial plant species richness slightly reduced the relative number of predator species but dramatically shifted the ratio of predator to herbivore individuals (Haddad et al., 2009).

Although many invasive species threaten biodiversity and ecosystem functioning (Bax et al., 2003; Guy‐Haim et al., 2018; Maggi et al., 2015; Schaffelke et al., 2006), we did not find differences between the native and nonnative *Gracilaria* species across the consumer responses (Figure 2) or community composition (Figure 3) in either monoculture or mixture. In fact, the nonnative *Gracilaria* species even appeared to slightly, albeit nonsignificantly, outperform the native *Gracilaria* species in both monoculture and mixture (Figure 2). The absence of statistically significant differences observed here is consistent with recent studies, indicating a considerable degree of similarity in morphological complexity, palatability, chemical defenses, and the food and habitat preferences of epifaunal invertebrates between these congeneric *Gracilaria* species (Berke et al., 2020; Navarro‐Barranco et al., 2019; Thomsen et al., 2014) and, more broadly, the overall idiosyncratic influence of introduced marine foundation species on ecosystem functioning (Guy‐Haim et al., 2018). Nevertheless, given the long history of biodiversity and invasibility (Stachowicz et al., 1999), understanding how established invaders integrate into biodiversity–ecosystem functioning relationships is a key frontier worthy of further exploration, especially as species' ranges continue to shift under climate change (Doney et al., 2012).

For logistical reasons our simplistic design did not account for several important factors, including the prevalence of strong top‐down forces and widespread omnivory, that are known to play a key role in the organization and functioning of this hard‐substratum benthic community (Bruno & O'Connor, 2005; Douglass et al., 2008; Duffy & Hay, 2000; Moran et al., 2010; O'Connor & Bruno, 2007) and, more generally, marine ecosystems (Duffy et al., 2007; Thompson et al., 2007). As a result, we are unable to truly discern whether invertebrate colonists segregated among macroalgal habitats due to (i) an increase in the overall quantity of available food resources (Best et al., 2014; Duffy & Hay, 1994); (ii) an overall increase in food quality resulting from trade‐offs between nutritional content, palatability, plant chemical defenses, and associated epiphytes (Erickson et al., 2006; Hay et al., 1987, 1988); (iii) species‐ or functional group–specific host, habitat, or feeding preferences (Hay et al., 1988; Steneck & Dethier, 1994); (iv) an increase in the overall value of macroalgal habitats in providing refugia from predators and physical stress as a result of differences in macroalgal morphology and structural complexity (Angelini et al., 2011; Gan et al., 2019; Moran et al., 2010; Steneck & Dethier, 1994); or (v) some combination that optimizes complex trade‐offs among the factors described previously (Callaway et al., 2005; Duffy & Hay, 1994; Machado, Ferreira, & Leite, 2019; Mattila et al., 2008). Future explorations, ideally in combination with feeding and other choice assays, will be necessary to fully disentangle the mechanisms at play to explain our results.

Human practices, such as shoreline development, overfishing, and global shipping, are driving rapid and often irreversible losses and gains of species that provide the foundation for coastal habitats (Bax et al., 2003; Jackson et al., 2001; Waycott et al., 2009). Our results demonstrate that nonrandom changes in foundation species diversity can have cascading effects that alter the standing biomass and biotic structure of higher trophic levels in marine communities. Because the small herbivorous invertebrates examined here in turn compose the diet of larger benthic and pelagic predators such as fishes and crustaceans, shifts in foundation species diversity likely have nontrivial consequences for the functioning and dynamics of coastal food webs and the provisioning of goods and services, such as fisheries, on which many people depend (Duffy, 2006; Machado, Ferreira, Bueno, et al., 2019; Wong et al., 2011). More broadly, these findings indicate that several basic mechanisms underlying terrestrial plant biodiversity effects also operate in marine macrophyte‐based systems, including those made more speciose by nonnatives. Thus, although some plant species are certainly more productive than others, more plant species are almost always more productive than fewer for individual functions, not to mention the multitude of other functions that these ecosystems sustain (i.e., multifunctionality sensu Lefcheck et al., 2015), such that perhaps these “key” species should be preferentially conserved, while also managing for biodiversity in general as an added insurance policy, as has been suggested for fishes (Clare et al., 2022; Topor et al., 2019). We argue that an enhanced understanding of the effects of realistic changes in foundation species diversity, as we present here, is increasingly vital to our ability to predict the ramifications of human‐driven extinctions and invasions and, ultimately, guide sound policies and decisions that will secure the sustainable future of marine fisheries and ecosystems in our rapidly changing world.

## AUTHOR CONTRIBUTIONS

Aaron P. Ramus and Zachary T. Long designed the experiment and analyzed data. Aaron P. Ramus performed field research, laboratory assays, and data collection. Jonathan S. Lefcheck and Aaron P. Ramus analyzed output data. Aaron P. Ramus, Jonathan S. Lefcheck, and Zachary T. Long conceptualized the paper, Aaron P. Ramus and Zachary T. Long drafted the manuscript, and Jonathan S. Lefcheck contributed substantially to revisions. All authors commented on and edited the manuscript and gave final approval for publication.

## CONFLICT OF INTEREST

The authors declare no conflict of interest.

## Supporting information


Appendix S1

## Data Availability

Data and code (Ramus et al., 2022) are available on Zenodo: https://doi.org/10.5281/zenodo.6588537.
