# Supplementary material for: Foundational biodiversity effects propagate through coastal food webs via multiple pathways
Source: Ecology. 2022 Jul 27;103(11):e3796. doi: 10.1002/ecy.3796 (PMC9787374; doi:10.1002/ecy.3796)
Supplement: Supplementary file 1 — Appendix S1 [file ECY-103-e3796-s001.pdf]

Supporting Information. Ramus, A. P., J. S. Lefcheck, and Z. T. Long. 2022. Foundational biodiversity effects propagate through coastal food webs via multiple pathways. Ecology.

Appendix S1

Table S1. Estimates of regression coefficients and their standard errors (SE) from the structural equation model (Fig. 1). Standardized coefficients are shown in bold when significant ($P < 0.05$).

Response	Predictor	Estimate	SE	DF	t-value	P-value	Std. Estimate
Macroalgal wet mass	Macroalgal richness	0.064	0.022	127	2.861	**0.005**	**0.243**
Invertebrate biomass	Macroalgal richness	0.011	0.017	124	0.617	0.538	0.025
Invertebrate biomass	Macroalgal wet mass	0.380	0.077	124	4.916	**<0.001**	**0.234**
Invertebrate biomass	Invertebrate richness	0.980	0.158	124	6.189	**<0.001**	**0.299**
Invertebrate biomass	Invertebrate abundance	0.005	0.000	124	9.344	**<0.001**	**0.481**
Invertebrate richness	Macroalgal richness	0.026	0.010	125	2.724	**0.007**	**0.202**
Invertebrate richness	Macroalgal wet mass	0.093	0.043	125	2.186	**0.031**	**0.188**
Invertebrate richness	Invertebrate abundance	0.001	0.000	125	4.412	**<0.001**	**0.380**
Invertebrate abundance	Macroalgal richness	3.542	3.068	126	1.155	0.250	0.086
Invertebrate abundance	Macroalgal wet mass	80.585	11.830	126	6.812	**<0.001**	**0.509**

Table S2. Pairwise planned contrasts for the effects of macroalgal treatment on invertebrate community composition (Fig. 3). Significant ($P < 0.05$) results from pairwise tests between treatments are bolded.

Contrast	F-value	R^2	P-value
Gracilaria tikvahiae - *Codium fragile*	9.16	0.22	**0.01**
Gracilaria vermiculophylla - *Codium fragile*	14.89	0.31	**0.01**
Gymnogongrus griffithsiae - *Codium fragile*	26.05	0.44	**0.01**
3 species native - *Codium fragile*	19.07	0.37	**0.01**
3 species nonnative - *Codium fragile*	23.95	0.42	**0.01**
4 species - *Codium fragile*	18.57	0.36	**0.01**
Gracilaria vermiculophylla - *Gracilaria tikvahiae*	1.34	0.04	0.20
Gymnogongrus griffithsiae - *Gracilaria tikvahiae*	6.49	0.16	**0.02**
3 species native -*Gracilaria tikvahiae*	2.75	0.08	**0.05**
3 species nonnative - *Gracilaria tikvahiae*	4.55	0.12	**0.03**
4 species - *Gracilaria tikvahiae*	2.16	0.06	0.12
Gymnogongrus griffithsiae - *Gracilaria vermiculophylla*	3.23	0.09	**0.05**
3 species native - *Gracilaria vermiculophylla*	1.05	0.03	0.33
3 species nonnative - *Gracilaria vermiculophylla*	1.54	0.04	0.18
4 species - *Gracilaria vermiculophylla*	0.76	0.02	0.47
3 species native - *Gymnogongrus griffithsiae*	1.13	0.03	0.36
3 species nonnative - *Gymnogongrus griffithsiae*	0.31	0.01	0.75
4 species - *Gymnogongrus griffithsiae*	0.91	0.03	0.44
3 species nonnative - 3 species native	0.57	0.02	0.63
4 species - 3 species native	0.23	0.01	0.86
4 species - 3 species nonnative	0.33	0.01	0.82

Table S3. Results of *t*-tests analyzing whether components of the biodiversity effect (Fig. 4) differ from zero in each treatment. Probability values appear in bold when significant ($P < 0.05$).

Response	Component	Treatment	t-value	DF	P-value
Invertebrate abundance (#)	Complementarity effect	3 species native	2.691	4	0.055
Invertebrate abundance (#)	Complementarity effect	3 species nonnative	5.522	4	**0.005**
Invertebrate abundance (#)	Complementarity effect	4 species	1.694	4	0.166
Invertebrate abundance (#)	Selection effect	3 species native	0.715	4	0.514
Invertebrate abundance (#)	Selection effect	3 species nonnative	0.674	4	0.537
Invertebrate abundance (#)	Selection effect	4 species	1.445	4	0.222
Invertebrate abundance (#)	Net biodiversity effect	3 species native	2.444	4	0.071
Invertebrate abundance (#)	Net biodiversity effect	3 species nonnative	11.159	4	**<0.001**
Invertebrate abundance (#)	Net biodiversity effect	4 species	2.433	4	0.072
Invertebrate biomass (g)	Complementarity effect	3 species native	3.119	4	**0.036**
Invertebrate biomass (g)	Complementarity effect	3 species nonnative	3.474	4	**0.025**
Invertebrate biomass (g)	Complementarity effect	4 species	1.115	4	0.327
Invertebrate biomass (g)	Selection effect	3 species native	-1.302	4	0.263
Invertebrate biomass (g)	Selection effect	3 species nonnative	-0.516	4	0.633
Invertebrate biomass (g)	Selection effect	4 species	0.505	4	0.640
Invertebrate biomass (g)	Net biodiversity effect	3 species native	0.229	4	0.830

Table S3 (continued)

Response	Component	Treatment	t-value	DF	P-value
Invertebrate biomass (g)	Net biodiversity effect	3 species nonnative	1.820	4	0.143
Invertebrate biomass (g)	Net biodiversity effect	4 species	4.248	4	**0.013**
Invertebrate richness (# of taxa)	Complementarity effect	3 species native	17.879	4	**<0.001**
Invertebrate richness (# of taxa)	Complementarity effect	3 species nonnative	11.647	4	**<0.001**
Invertebrate richness (# of taxa)	Complementarity effect	4 species	12.227	4	**<0.001**
Invertebrate richness (# of taxa)	Selection effect	3 species native	-1.741	4	0.157
Invertebrate richness (# of taxa)	Selection effect	3 species nonnative	-0.960	4	0.392
Invertebrate richness (# of taxa)	Selection effect	4 species	-0.414	4	0.700
Invertebrate richness (# of taxa)	Net biodiversity effect	3 species native	31.348	4	**<0.001**
Invertebrate richness (# of taxa)	Net biodiversity effect	3 species nonnative	13.208	4	**<0.001**
Invertebrate richness (# of taxa)	Net biodiversity effect	4 species	17.575	4	**<0.001**
Macroalgal wet mass (g)	Complementarity effect	3 species native	10.368	4	**<0.001**
Macroalgal wet mass (g)	Complementarity effect	3 species nonnative	2.157	4	0.097
Macroalgal wet mass (g)	Complementarity effect	4 species	4.003	4	**0.016**
Macroalgal wet mass (g)	Selection effect	3 species native	13.725	4	**<0.001**
Macroalgal wet mass (g)	Selection effect	3 species nonnative	1.751	4	0.155
Macroalgal wet mass (g)	Selection effect	4 species	0.434	4	0.687
Macroalgal wet mass (g)	Net biodiversity effect	3 species native	14.280	4	**<0.001**

Table S3 (continued)

Response	Component	Treatment	t-value	DF	P-value
Macroalgal wet mass (g)	Net biodiversity effect	3 species nonnative	2.196	4	0.093
Macroalgal wet mass (g)	Net biodiversity effect	4 species	3.097	4	**0.036**

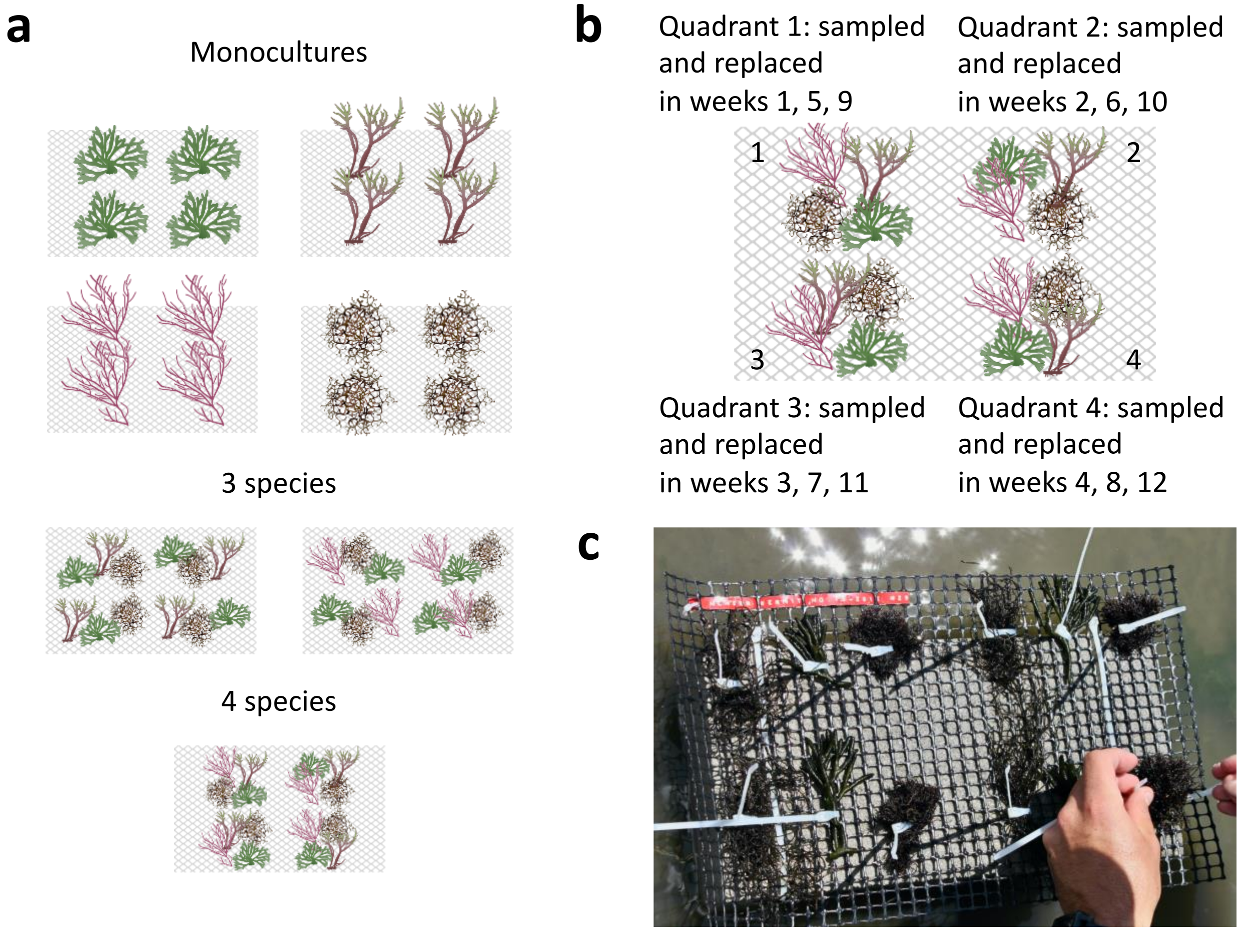

Figure S1. Schematic diagram of the experiment and sampling design. In (a), summary of the seven macroalgal treatments, representing 3 levels of macroalgal richness, following a substitutive design (i.e., total wet mass is held constant in each quadrant); (b) sampling procedure used over the course of the experiment for the weekly destructive sampling of the macroalgal treatments, replicated within each quadrant of each block; (c) picture showing an experimental block containing a 3 species nonnative treatment in each quadrant.

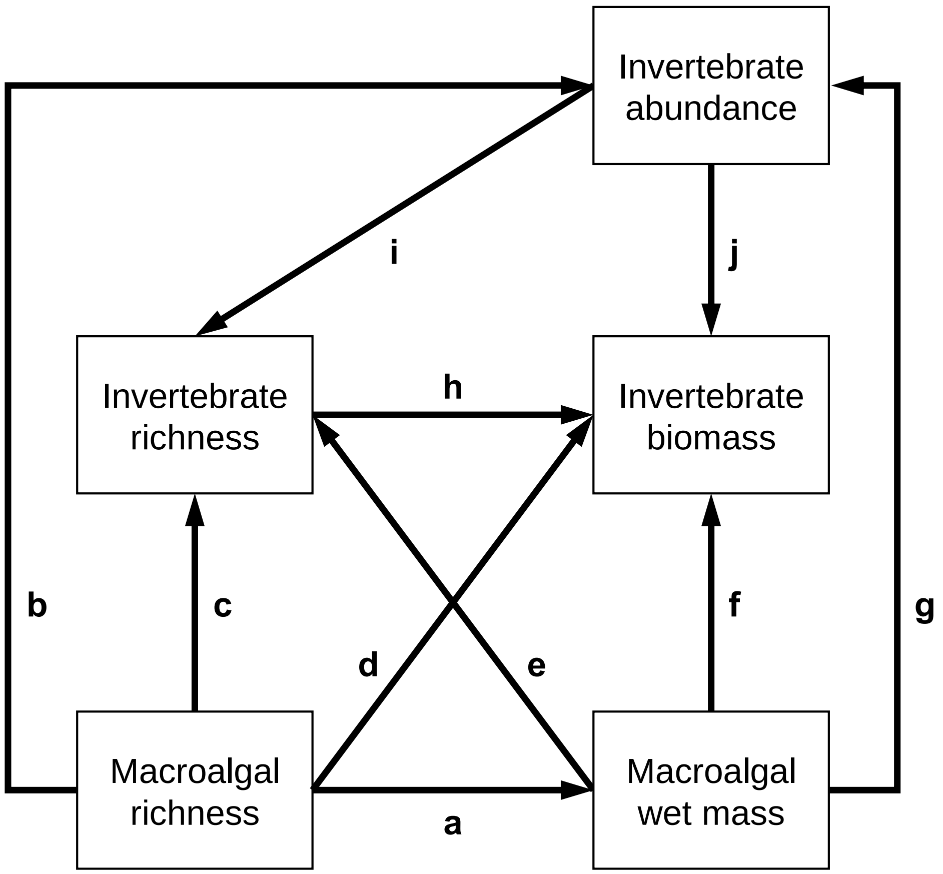

Figure S2. Conceptual representation and justification of linkages in our hypothesized causal model. We expected that: (a) macroalgal richness increases macroalgal wet mass (Bruno et al. 2005, 2006, Stachowicz et al. 2008, Boyer et al. 2009); (b, c, and d) macroalgal richness increases invertebrate richness, biomass, and/or abundance through provisioning of unique habitats and/or food resources (Siemann et al. 1998, Haddad et al. 2009, Borer et al. 2012, Ebeling et al. 2014, 2018, Hertzog et al. 2016, Schuldt et al. 2019); (e, f, and g) macroalgal wet mass increases invertebrate richness, biomass, and/or abundance through provisioning of habitat and/or food writ large (Scherber et al. 2010, Borer et al. 2012, Ebeling et al. 2014, 2018, Hertzog et al. 2016); (h) invertebrate richness increases invertebrate biomass (Duffy et al. 2003, 2005); (i) invertebrate abundance increases invertebrate richness (Ebeling et al. 2014, Hertzog et al. 2016, Schuldt et al. 2019); and (j) invertebrate abundance increases invertebrate biomass, as adding individuals necessarily must add biomass.

LITERATURE CITED

Borer, E. T., E. W. Seabloom, and D. Tilman. 2012. Plant diversity controls arthropod biomass and temporal stability. Ecology Letters 15:1457–1464.

Boyer, K. E., J. S. Kertesz, and J. F. Bruno. 2009. Biodiversity effects on productivity and stability of marine macroalgal communities: The role of environmental context. Oikos 118:1062–1072.

Bruno, J. F., K. E. Boyer, J. E. Duffy, S. C. Lee, and J. S. Kertesz. 2005. Effects of macroalgal species identity and richness on primary production in benthic marine communities. Ecology Letters 8:1165–1174.

Bruno, J. F., S. C. Lee, J. S. Kertesz, R. C. Carpenter, Z. T. Long, and J. E. Duffy. 2006. Partitioning the effects of algal species identity and richness on benthic marine primary production. Oikos 115:170–178.

Duffy, J. E., J. P. Richardson, and E. A. Canuel. 2003. Grazer diversity effects on ecosystem functioning in seagrass beds. Ecology Letters 6:637–645.

Duffy, J. E., J. P. Richardson, and K. E. France. 2005. Ecosystem consequences of diversity depend on food chain length in estuarine vegetation. Ecology Letters 8:301–309.

Ebeling, A. et al. 2014. Plant diversity impacts decomposition and herbivory via changes in aboveground arthropods. PLoS ONE 9:e106529.

Ebeling, A., J. Hines, L. R. Hertzog, M. Lange, S. T. Meyer, N. K. Simons, and W. W. Weisser. 2018. Plant diversity effects on arthropods and arthropod-dependent ecosystem functions in a biodiversity experiment. Basic and Applied Ecology 26:50–63.

Haddad, N. M., G. M. Crutsinger, K. Gross, J. Haarstad, J. M. H. Knops, and D. Tilman. 2009. Plant species loss decreases arthropod diversity and shifts trophic structure. Ecology Letters 12:1029–1039.

Hertzog, L. R., S. T. Meyer, W. W. Weisser, and A. Ebeling. 2016. Experimental manipulation of grassland plant diversity induces complex shifts in aboveground arthropod diversity. PLoS ONE 11:e0148768.

Scherber, C. et al. 2010. Bottom-up effects of plant diversity on multitrophic interactions in a biodiversity experiment. Nature 468:553–556.

Schuldt, A. et al. 2019. Multiple plant diversity components drive consumer communities across ecosystems. Nature Communications 10:1460.

Siemann, E., D. Tilman, J. Haarstad, and M. Ritchie. 1998. Experimental tests of the dependence of arthropod diversity on plant diversity. The American Naturalist 152:738–750.

Stachowicz, J. J., M. Graham, M. E. S. Bracken, and A. I. Szoboszlai. 2008. Diversity enhances cover and stability of seaweed assemblages: The role of heterogeneity and time. Ecology 89:3008–3019.
